# Retrospective Review of the Anaesthetic Management of Maxillectomies and Mandibulectomies for Benign Tumours in Sub-Saharan Africa

**DOI:** 10.1371/journal.pone.0165090

**Published:** 2016-10-27

**Authors:** Michelle C. White, Katherine C. Horner, Peggy S. Lai

**Affiliations:** 1 Department of Anaesthesia, Mercy Ships, Port au Toamasina, Region 12, Madagascar; 2 Division of Pulmonary and Critical Care, Massachusetts General Hospital, Boston, United States of America; University of Colorado, UNITED STATES

## Abstract

**Background:**

Safe anaesthesia is a crucial component of safe surgical care, yet anaesthetic complications are common in resource-limited settings. We describe differences in anaesthetic needs for Mandibulectomy vs. Maxillectomy in three sub-Saharan African countries.

**Materials and Methods:**

Retrospective review of patients undergoing minor Mandibulectomy, major Mandibulectomy, or Maxillectomy in Togo, Guinea and Republic of the Congo. Surgeries were performed on the *Africa Mercy*, an international non-governmental hospital ship. Primary outcomes were need for advanced airway management and intra-operative blood loss. Secondary outcomes were time under general anaesthesia and hospital length of stay. Multivariate regression determined the association between operation type and each outcome measure.

**Results:**

105 patients were included (25 minor Mandibulectomy, 58 major Mandibulectomy, 22 Maxillectomy procedures). In-hospital mortality was 0%. 44/105 (41.9%) required an advanced airway management technique to achieve intubation, although in all cases this was anticipated prior to the procedure; no differences were noted between surgical procedure (p = 0.72). Operative procedure was a significant risk factor for intra-operative blood loss. Patients undergoing Maxillectomy lost on average 851.5 (413.3, 1289.8, p = 0.0003) mL more blood than patients undergoing minor Mandibulectomy, and 507.3 (150.3, 864.3, p = 0.007) mL more blood than patients undergoing major Mandibulectomy. Patients undergoing Maxillectomy had a significantly higher time under general anaesthesia than those undergoing minor Mandibulectomy. There was no significant difference in hospital length of stay between operation type.

**Conclusion:**

Anaesthetic considerations for minor Mandibulectomy, major Mandibulectomy, and Maxillectomy differ with respect to intra-operative blood loss and time under general anaesthesia, but not need for advanced airway management or length of stay. Although advanced airway management was required in 41.9% of patients, there were no unanticipated difficult airways. With appropriate training and resources, safe anaesthesia can be delivered to patients from low-income countries requiring major head and neck surgery.

## Introduction

Safe anaesthesia is an essential component of safe surgical care, yet death from anaesthesia remains significantly higher in low and middle-income countries (LMICs) compared to high-income countries [1]. Studies from Malawi [2], Zimbabwe[3], and Togo [4] report rates of anaesthetic mortality ranging from one death per 133 to 504 cases, whereas in high income countries anaesthetic mortality is 34 per million anaesthetics administered [5]. Much of the discrepancy is attributable to lack of monitoring equipment, essential medications, and inadequate anaesthetic training or supervision in LMICs [6]. Another factor is the health of the patient. Malnutrition and anaemia are common [7], as well as late presentation, with benign head and neck tumours growing to huge sizes that complicate the anaesthetic approach [8, 9]. Given the number of factors involved, it is often difficult to identify the key factors associated with anaesthetic complications in resource-limited settings.

Several small case series report the surgical difficulties associated with major head and neck surgery in resource-limited settings, but there is little published data describing anaesthetic management or complications [10]. A single study from Nigeria reports a 9% anaesthesia-related mortality for major head and neck surgery [8]. Disentangling the factors associated with this high mortality is difficult. Many hospitals in resource-limited settings may lack the anaesthetic training to identify which cases can be safely performed given the available anaesthetic expertise or equipment. Furthermore, while anaesthetic providers from high-income countries may have significant clinical training, they are unlikely to have encountered tumours of the size encountered in resource-limited settings [9].

Mercy Ships is a non-governmental organization that provides free surgical care in Africa on board the *Africa Mercy* hospital ship. The *Africa Mercy* is a self-contained mobile surgical unit, with five operating rooms and 80 inpatient beds, as well as support services including a blood bank, clinical laboratory, radiology department and pharmacy. The ship docks for 10 months in each country, providing free elective surgery as well as post-operative care, outpatient follow up and rehabilitation. At any one time, the ship is staffed by up to 400 international volunteer crew. Patients are selected through screenings advertised nationwide via radio, newspaper, or through word of mouth; additionally, local providers provide a referral base. Part of the surgical infrastructure includes trained anaesthesia providers from high-income countries using advanced airway and monitoring equipment. This unique setup allows us to disentangle the elements of training and infrastructure from patient-related factors.

The aim of this study is to report our experience of the anaesthetic management of Maxillectomy and Mandibulectomy in patients from low-income countries, in order to help other providers anticipate anaesthetic needs in the peri-operative setting. We hypothesized that anaesthetic needs would differ based on the type of surgical procedure (minor Mandibulectomy, major Mandibulectomy, and Maxillectomy).

## Materials and Methods

Study data were obtained by retrospective review of all patients ≥ 14 years of age undergoing Maxillectomy or Mandibulectomy on board the *Africa Mercy* hospital ship between January 2012 and June 2014. During this time the ship was operating in Togo, Guinea and the Republic of the Congo, which rank 166^th^, 179^th^, and 140^th^ (out of 187 countries), respectively, on the United Nations Human Development Index [11].

The *Africa Mercy* anaesthesia department consists of the Anaesthesia Supervisor and approximately 70 short-term volunteers per 10-month period. Short-term volunteers have an average length of stay of 2 and 3 weeks for physician and nurse anaesthetists respectively. The Anaesthesia Supervisor is a full time British consultant anaesthetist and paediatric intensive care physician (equivalent to a United States (US) board-certified anaesthesiologist and paediatric intensivist). Anaesthesia is either administered by board-certified anaesthesiologists (or equivalent for non-US countries) or occasionally nurse anaesthetists supervised by the Anaesthesia Supervisor.

All patients are seen pre-operatively by a hospital physician and undergo a history and physical examination. According to hospital protocol, Lee’s Revised Cardiac Risk Index [12] is calculated for any patient with cardiopulmonary symptoms or previously undiagnosed hypertension. In high-income settings, the Revised Cardiac Risk Index classifies patients as Class 1, 2, 3 or 4 and estimates the risk of a major peri-operative cardiac event as 0.4%, 0.9%, 6.6% and 11% respectively. In our context, all Class 3 patients are referred to the Anaesthesia Supervisor and the risk/benefit of surgery discussed at a pre-operative multi-disciplinary meeting. All Class 4 patients are cancelled.

All patients have an anaesthesia pre-operative assessment the day before surgery by the attending anaesthesiologist and airway management decision-making is physician-led. If any airway management difficulties are suspected, the Anaesthesia Supervisor is consulted for a final decision on method of intubation and is available to supervise use of difficult airway equipment and induction of anaesthesia. Department intra-operative transfusion guidelines suggest a transfusion threshold of haemoglobin 8g/dL or 9–10 g/dL if there is ongoing blood loss and more than 500 mL is expected to be lost. The use of invasive monitoring is at the clinical discretion of the Anaesthesia Supervisor and reserved for patients with a Revised Cardiac Risk Index Class 2 or 3. All decisions involving admission to the intensive care unit (ICU) (both elective and emergency) are discussed with the Anaesthesia Supervisor who is also in charge of the ICU. Other decisions such as specific anaesthesia agents administered, mode of analgesia, and fluid administration are left to the clinical judgement and personal preference of the anaesthesia provider. Goal-directed fluid-management is encouraged according to clinical signs of heart rate, blood pressure and pulse pressure variation according to estimates from pulse oximeter plethysmography, but is not protocolized, and no non-invasive cardiac-output monitors are available.

Post-operative care is provided by fully qualified volunteer nurses working under the direction of the surgeons, anaesthesia providers, hospital physicians and experienced Mercy Ships nurse team leaders, on a ward with a ratio of 1 nurse to 4–6 patients (and 1 per 7–8 patients at night) depending on acuity. Outpatient follow-up and wound care is provided by Mercy Ships for up to 10 months if required.

Collected data included age, gender, weight, pre-operative haemoglobin value, American Society of Anaesthesiology (ASA) grade, type of surgery, airway management technique, time under general anaesthesia, estimated blood loss, requirement for intra- or post-operative blood transfusion, volume of blood transfused, volume of crystalloid infused, hospital length of stay, post-operative complications, and tumour histopathology. We used standard definitions of ASA score[13]. All patients with a Revised Cardiac Risk Index Class 3, were said to have an ASA score of 3.

Operation type was categorized into minor Mandibulectomy, major Mandibulectomy, and Maxillectomy. Major Mandibulectomy comprised of hemi-Mandibulectomy, subtotal Mandibulectomy and total Mandibulectomy. Minor Mandibulectomy comprised of segmental Mandibulectomy and anterior Mandibulectomy. All surgery was performed on board the *Africa Mercy*. Maxillectomy involved temporalis muscle flap reconstruction. Mandibulectomy involved titanium plate reconstruction with iliac crest bone grafting 3 months postoperatively—iliac crest bone grafting cases were not included in this analysis. Tumours of large size and long duration are assumed to be benign and not usually biopsied unless clinically indicated or radiographic imaging is suggestive of malignancy. Clinical indications for biopsy include rapidly growing lesion (larger than the size of a fist with a history of under 1–2 years), or fungating lesion. Malignant lesions are excluded from surgery.

Airway management technique was categorised into “Conventional”, meaning tracheal intubation was achieved via direct laryngoscopy with a traditional laryngoscope, or “Advanced” meaning tracheal intubation was achieved using a fibreoptic scope or video laryngoscope.

The primary outcomes of interest were need for an advanced airway management technique and estimated intra-operative blood loss. Secondary outcomes of interest were time under general anesthesia and hospital length of stay (LOS). This study did not intend to evaluate the impact of surgery on patient quality of life or life expectancy.

Summary statistics were calculated as mean ± standard deviation for continuous variables, with the exception of estimated blood loss, which was reported as median [interquartile range] as 78.5% of patients did not receive a blood transfusion. For binary or categorical variables, they are reported as number (percentage).

The primary exposure of interest was type of surgical procedure (minor Mandibulectomy, major Mandibulectomy, and Maxillectomy). In unadjusted analyses, either the Kruskall-Wallis test (for continuous outcomes) or the Fisher’s exact test (for binary outcomes) was used to test differences between demographic variable or outcome across operative procedure.

In adjusted analyses, linear regression (for continuous outcomes) or logistic regression (for binary outcomes) was used to determine the association between covariates and each outcome measure. The primary exposure of interest (operative procedure) was treated as a categorical variable with minor Mandibulectomy being the reference group. Covariates included age, male gender, weight, ASA status, and pre-operative hemoglobin. Continuous covariates were centered at the mean to make the intercept interpretable.

108 subjects were initially identified. One subject who underwent Maxillectomy had an estimated intra-operative blood loss of 10,000 mL; this outlier value was excluded so as not to skew results for the outcome of estimated blood loss and time under general anaesthesia. Two subjects did not have ASA status recorded pre-operatively and were also excluded, resulting in 105 subjects for the final analysis.

All analyses were performed using R 3.2.2. Two sided p-values of <0.05 were considered significant.

Author MCW was the Mercy Ships Anesthesia Supervisor and was involved in most, but not all of the patients’ medical treatment. Individual patient consent was not obtained for this retrospective review but all data were anonymized and de-identified by the author MCW prior to analysis. Approval for the study (including not requiring individual patient consent for adults and children) was obtained from the Mercy Ships Institutional Review Board (protocol numbers MS-2014-01 and MS-2014-02).

## Results

Baseline characteristics of study patients, stratified by operative procedure, are as displayed in Table 1. 25, 58, and 22 patients with facial tumours underwent minor Mandibulectomy, major Mandibulectomy, and Maxillectomy respectively. The average age of participants was 31.4 ± 12.2 years; only 1 patient was less than 18 years of age. 50.5% were male in the overall cohort. 55 (52.4%), 40 (38.1%), and 10 (9.5%) had ASA grades of I, II, and III respectively. Mean pre-operative haemoglobin was 12.7 ± 2.2 g/dL. There were no significant differences in age (p = 0.51), gender (p = 0.10), weight (p = 0.46), pre-operative haemoglobin (p = 0.69), or ASA status (p = 0.16) across operative procedure.

**Table 1:** Baseline characteristics of study subjects stratified by operation type. Values reported as n (%) or mean ± sd. Blood administered reported as median [interquartile range] as most (78.5%) patients did not receive a blood transfusion. There were no significant differences in age (p = 0.51), gender (p = 0.10), weight (p = 0.46), pre-operative haemoglobin (p = 0.69), or ASA status (p = 0.16) across operative procedure.

	Mandibulectomy (minor)	Mandibulectomy (major)	Maxillectomy	All
Observations (n)	25	58	22	105
Age (years)	29.2 ± 10.8	32.5 ± 12.5	31.1 ± 13.2	31.4 ± 12.2
Men	8 (32.0%)	32 (55.2%)	13 (59.1%)	53 (50.5%)
Weight (kilograms)	63.3 ± 12.7	61.5 ± 11.5	58.7 ± 18.0	61.4 ± 13.3
Pre-operative haemoglobin (g/dL)	12.3 ± 2.4	12.8 ± 2.1	12.7 ± 2.1	12.7 ± 2.2
ASA Classification				
1	17 (68.0%)	29 (50.0%)	9 (40.9%)	55 (52.4%)
2	8 (32.0%)	23 (39.7%)	9 (40.9%)	40 (38.1%)
3	0 (0%)	6 (10.3%)	4 (18.2%)	10 (9.5%)
Hospital Course				
Fluid administered (mL)	1688.0 ± 472.9	2249.0 ± 928.8	3186.4 ± 1295.9	2311.8± 1058.8
Blood administered (mL)	0 [0–0]	0 [0–0]	225.0 [0–450.0]	0 [0–0]
Pathologic diagnosis				
Ameloblastoma	19 (76.0%)	48 (82.8%)	4 (18.2%)	71 (67.6%)
Ossifying fibroma	3 (12.0%)	2 (3.4%)	0 (0%)	5 (4.8%)
Fibrous dysplasia	1 (4.0%)	0 (0%)	3 (13.6%)	4 (3.8%)
Other	2 (8.0%)	8 (13.8%)	15 (68.2%)	25 (23.8%)

In-hospital mortality was 0%. Only 21.5% of patients received a blood transfusion in the intra- or post-operative period. 2/105 (1.9%) had invasive arterial monitoring and no patients had central venous pressure monitoring or required inotropic support. No patient had an unexpected difficult intubation (as predicted by standard pre-operative anaesthesia assessment); all received intra-operative dexamethasone and all were successfully extubated at the end of surgery. One had a post-operative bleed 12 hours after surgery resulting in airway obstruction. This patient required emergency surgery and remained intubated and ventilated for 3 days while airway oedema resolved. The most common pathologic diagnosis was ameloblastoma in 71 (67.6%) subjects overall and in 19 (76.0%), 48 (82.8%), and 4 (18.2%) subjects undergoing minor Mandibulectomy, major Mandibulectomy, and Maxillectomy.

In unadjusted analyses (Table 2), the main difference in outcomes between operation types was estimated intra-operative blood loss and time under general anaesthesia. Average blood loss was 426.0 ± 221.8 mL, 846.6 ± 772.0 mL, and 1422.7 ± 1016.8 mL in minor Mandibulectomy, major Mandibulectomy, and Maxillectomy patients respectively (p < 0.001). The average time under general anaesthesia was 229.2 ± 47.7, 277.4 ± 96.6, and 401.8 ± 118.2 minutes for minor Mandibulectomy, major Mandibulectomy, and Maxillectomy respectively (p < 0.001). Although 44/105 (41.9%) patients required advanced airway management, there were no significant differences in this requirement between operative procedure (p = 0.72). Similarly, there were no differences in average hospital length of stay (p = 0.22).

**Table 2:** Unadjusted outcomes stratified by operation type. Comparison of outcomes between groups in crude data shows no significant differences in need for advanced airway management (p = 0.72) or hospital length of stay (LOS, p = 0.22), but significant differences in estimated blood loss (p < 0.001) and time under general anaesthesia (p < 0.001).

	Mandibulectomy (minor)	Mandibulectomy (major)	Maxillectomy	All
Observations (n)	25	58	22	105
Airway				
Conventional	15 (60.0%)	35 (60.3%)	11 (50.0%)	61 (58.1%)
Advanced ^a^	10 (40.0%)	23 (39.7%)	11 (50.0%)	44 (41.9%)

Estimated blood loss (mL)	426.0 ± 221.8	846.6 ± 772.0	1422.7 ± 1016.8	867.1 ± 811.8
Time under general anaesthesia (min)	229.2 ± 47.7	277.4 ± 96.6	401.8 ± 118.2	292.0 ± 109.9
Hospital LOS (days)	8.4 ± 2.2	9.2 ± 4.3	10.1 ± 6.9	9.2 ± 4.6

^a^ Advanced airway defined as need for video laryngoscopy or fibreoptic intubation.

These differences in outcomes across operative procedure persisted in multivariate analyses (Tables 3 and 4). Maxillectomies had significantly higher estimated intra-operative blood loss than both major Mandibulectomy (507.3 (150.3, 864.3) mL, p = 0.007) and minor Mandibulectomy (851.5 (413.3, 1289.8) mL, p = 0.0003); differences in major vs. minor Mandibulectomy did not reach statistical significance (344.2 (-11.1, 699.6) mL, p = 0.061). Likewise, time under general anaesthesia was higher for patients undergoing Maxillectomy as compared to major Mandibulectomy (117.2 (72.2, 162.2) minutes longer for Maxillectomy, p = 0.00001) and minor Mandibulectomy (161.1 (105.9, 216.4) minutes longer for Maxillectomy, p < 0.00001). Differences between major and minor Mandibulectomy did not reach statistical significance (43.9 (-0.8, 88.7) minutes longer for major vs. minor Mandibulectomy, p = 0.058). There were no significant differences in need for advanced airway management or hospital length of stay across operative procedures.

**Table 3:** Predictors of primary outcomes (need for advanced airway management and estimated intra-operative blood loss). Estimated blood loss but not need for advanced airway management differed across procedure. Note for operative procedure (minor Mandibulectomy, major Mandibulectomy, Maxillectomy), minor Mandibulectomy served as the reference group. Intercept represents the average patient, i.e. 31.3 year old male patient with ASA I, weight of 61.1 kilograms, and pre-operative haemoglobin of 12.6 g/dL undergoing minor Mandibulectomy.

	Advanced Airway Management OR (95% CI)	Estimated blood loss mL (95% CI)
Age (years)	1.0 (1.0, 1.1)	0.8 (-11.9, 13.5)
Female	reference	reference
Male	0.9 (0.3, 2.4)	421.5** (76.1, 766.8)
Weight (kilograms)	1.0 (0.9, 1.0)	-3.3 (-15.9, 9.2)
ASA I	reference	reference
ASA II	0.5 (0.2, 1.2)	-147.4 (-459.6, 164.9)
ASA III	0.8 (0.2, 3.6)	382.8 (-123.5, 889.1)
Pre-operative haemoglobin (g/dL)	0.9 (0.7, 1.1)	-103.6** (-188.3, -18.9)
Minor Mandibulectomy	reference	reference
Major Mandibulectomy	1.1 (0.4, 3.2)	344.2* (-11.1, 699.6)
Maxillectomy	1.7 (0.5, 6.3)	851.5*** (413.3, 1,289.8)
Intercept	0.9 (0.3, 2.3)	314.7* (-14.6, 644.1)
Observations	105	105

*p<0.1;

**p<0.05;

***p<0.01

**Table 4:** Predictors of secondary outcomes (time under general anaesthesia and hospital length of stay). Time under general anaesthesia but not hospital length of stay was different across procedure. Note for operative procedure (minor Mandibulectomy, major Mandibulectomy, Maxillectomy), minor Mandibulectomy served as the reference group. Intercept represents the average patient, i.e. 31.3 year old male patient with ASA I, weight of 61.1 kilograms, and pre-operative haemoglobin of 12.6 g/dL undergoing minor Mandibulectomy.

	Time under general anaesthesia minutes (95% CI)	Hospital LOS days (95% CI)
Age (years)	-0.05 (-1.6, 1.6)	-0.03 (-0.1, 0.05)
Female	reference	reference
Male	13.1 (-30.4, 56.7)	-0.8 (-2.9, 1.4)
Weight (kilograms)	-0.03 (-1.6, 1.5)	-0.02 (-0.1, 0.1)
ASA I	reference	reference
ASA II	-22.5 (-61.9, 16.8)	-0.3 (-2.3, 1.6)
ASA III	71.5** (7.7, 135.3)	3.9** (0.7, 7.0)
Pre-operative haemoglobin (g/dL)	-7.8 (-18.5, 2.8)	-0.2 (-0.7, 0.3)
Minor Mandibulectomy	reference	reference
Major Mandibulectomy	43.9* (-0.8, 88.7)	0.7 (-1.5, 3.0)
Maxillectomy	161.1*** (105.9, 216.4)	1.3 (-1.5, 4.0)
Intercept	229.8*** (188.3, 271.3)	8.7*** (6.7, 10.8)
Observations	105	105

*p<0.1;

**p<0.05;

***p<0.01

## Discussion

To our knowledge, this is the first publication describing anaesthetic considerations for benign tumours requiring Mandibulectomy or Maxillectomy in patients from resource-limited settings. Our results suggest that blood loss may be high and varies by procedure, with patients undergoing Maxillectomy at the highest risk of major haemorrhage (mean blood loss 1422.7 mL). Difficult airway management requiring advanced intubation techniques such as fibreoptic intubation or video laryngoscopy, is common (41.9%) but predictable by standard pre-operative anaesthesia assessment when performed by trained anaesthetists.

Quality and performance indicators exist for head and neck surgery in high-income countries [14] and while our results suggest that transfusion requirements, complications and hospital length of stay compare favourably with western standards, the aetiology and patient demographics are very different. Our patient population is younger (mean age 31.4 years), fitter (90.5% ASA 1 or 2) and have non-malignant pathology. To a certain extent, this may account for the lack of need for invasive monitoring, inotropes, post-operative intensive care and for a shorter hospital length of stay (mean 9.2 days). These factors and our low incidence of blood transfusion may account for our zero mortality.

Estimated intra-operative blood loss is significantly greater in Maxillectomy (mean 1422.7 mL) compared with major and minor Mandibulectomy (mean 846.6 and 426.0 mL respectively). Anaesthesia providers should be prepared for this with adequate amounts of intravenous fluid and blood available; two large bore cannulae and good communication with the surgeon as recommended by the WHO Surgical Safety checklist [15]. Where an on-site blood bank does not exist, our results suggest that undertaking Maxillectomy could be hazardous due to the risk of major blood loss and need for transfusion.

Unexpected difficult intubation is a life-threatening event, even in high resource settings. In low resource settings where there is a lack of equipment and training to manage this situation, it is even more risky. Therefore, it is clinically significant that in our case series, despite a high requirement for advanced airway techniques (41.9%) there were no ‘unexpected’ difficult intubations. Airway complications are common in head and neck surgery and our lack of unexpected difficult intubations or failed extubations is likely explained by careful planning, the presence of an experienced anaesthetist, elective rather than emergency caseload, and relatively few obese patients compared to high income settings [16, 17]. Additionally, because of the extensive nature of the tumours in our study, all patients had a pre-operative computerised tomography (CT) scan. Although this may have aided airway assessment with regard to patency of the nares when nasal intubation was planned, CT scan does not predict the ability to visualise the larynx by direct laryngoscopy. Anaesthetists were therefore still required to use standard methods for airway assessment and the use of CT, while helpful, does not adequately explain our low incidence of unexpected difficult intubation. Our results suggest that despite extensive facial pathology, standard preoperative airway assessment may be sufficient to plan airway management. However, good decision-making remains crucial since the need for advanced airway management techniques is high (41.9%).

Anaesthesia decision-making in 3 keys areas is likely to have contributed to the success of our approach: airway management; estimating the Revised Cardiac Risk Index [12] and excluding high risk patients; fluid and blood management given that blood loss may be greater than 10% of the estimated circulating volume for Maxillectomies and major Mandibulectomies (mean 1422.7 mL and 846.6 mL respectively). Each of these areas do not require ‘high-tech’ equipment per se, or advanced anaesthesia skills, but rather careful attention to detail and vigilance. This should be highlighted to anaesthesia providers in LMICs intending to administer anaesthesia for Maxillectomy or Mandibulectomy.

In low-income countries, lack of advanced airway equipment need not be a rate-limiting step for performing Mandibulectomy or Maxillectomy. In our experience, in the countries in this study, surgeons routinely perform awake tracheostomy for patients undergoing other maxillofacial procedures such as ankylosis release. Awake tracheostomy would be a suitable alternative to the advanced airway management techniques we used (fibreoptic intubation and video laryngoscopy). Additionally, tracheostomy avoids the hazards of extubation and airway oedema in the immediate post-operative period. Blood loss and availability of a functioning blood bank is likely to be the more rate-limiting step for undertaking these procedures than availability of video laryngoscopes, fibreoptic scopes or other ‘advanced’ airway equipment. Anaesthesia providers who are ‘well trained in a western environment’ and come to assist in major cases in low resource settings may not be familiar with awake tracheostomy. A large prospective study of airway complications in the United Kingdom showed evidence that awake tracheostomy was not used on several occasions when it was the logical solution[16, 17]. Therefore western anaesthesia providers may not feel comfortable advocating awake tracheostomy, yet it is a widely used technique in low-income settings and should not be discounted.

Other anaesthetic requirements were “low-tech” compared with major maxillofacial surgery in western settings. Only 2/105 patients had invasive arterial pressure monitoring; none required central venous pressure monitoring or inotropic support; and all were extubated at the end of the surgery. This suggests that Maxillectomy and Mandibulectomy could be undertaken in the local hospital environment in low-income countries without the need for sophisticated equipment.

There was a high incidence of ameloblastoma in our study population. Ameloblastoma is commonly associated with unerupted teeth and is more common in black Africans than Caucasians [18, 19]. Initially ameloblastoma is a very small lesion easily dealt with in high-income settings by a dentist or oral surgeon and does not present as a large tumour requiring major head and neck surgery. However, in LMICs where affordable and accessible dental and surgical care is lacking, benign tumours such as ameloblastoma grow unabated to huge sizes, unlike malignant tumours, which quickly cause death. Ameloblastoma is therefore a common cause of advanced benign facial tumour presenting for treatment in LMICs, which explains the high incidence found in our study.

Our study has a few limitations. Surgery and anaesthesia were performed in the unique setting of a “high-tech” hospital ship using western-trained anaesthesia providers with experienced supervision. Although this is not immediately generalizable to hospitals in LMICs without trained providers, we feel that our findings remain useful as it demonstrates that safe anaesthesia for these complex surgeries is possible with appropriate training, and highlights areas of anaesthetic planning that should be taken into account prior to the start of these cases. High-risk patients and those with malignant tumours were excluded and may have introduced selection bias. A number of potentially confounding variables such as daily activity levels were not recorded and accounted for in the statistical analysis. Additionally ASA physical status, while commonly reported, is insensitive when used for risk stratification and using the Revised Cardiac Risk Index [12] would have been preferable. Although Mercy Ships has a protocol for calculating Revised Cardiac Risk Index, this is not done in every patient and therefore could not be used in our analysis. Finally, our sample size is too small to infer interpretations of ‘safety’ for rare events such as failed extubation.

Despite these limitations we believe our study has some unique strengths. Patient selection is a key component of safe surgery and our pragmatic decision to exclude high-risk patients and those with malignancy is directly relevant to a low-resource setting, where it is ethically questionable to perform major surgery on a patient without a reasonable expectation of success. This is particularly true when the patient and/or their family may face financial catastrophe due to the cost of surgery [20]. Our avoidance of invasive monitoring and post-operative intensive care, and clinically driven goal-directed fluid replacement means that the anaesthesia techniques used should be reproducible in LMICs. Also, our reported incidences of difficult intubation and blood loss should allow informed decisions to be made about whether to proceed with surgery and how much blood loss should be anticipated.

In summary, we suggest that anaesthesia for Maxillectomy and Mandibulectomy can be carried out in a low resource setting without the need for complex monitoring or airway equipment if performed by trained anaesthesia providers. Key features of anaesthetic management focus on decision-making for airway management and in the case of Maxillectomy, preparations for major blood loss to ensure blood is available for transfusion.
